# Buildings Contemplated

**Published:** 1914-09-26

**Authors:** 


### Buildings Contemplated.

Banbury.—The Rural District Council have instructed the surveyor to obtain particulars of a portable building suitable for an isolation hospital.

Barnet.—The Guardians have decided to obtain tenders for additions to the new infirmary.

Hereford.—The Rural District Council have agreed to apply to the Local Government Board for sanction to a loan of £3,600 for the extension of the isolation hospital.

Nuneaton.—The Guardians of Nuneaton Union are inviting tenders for the erection of children's homes. Mr. E. E. Shepherd, M.S.A., is the architect, from whom all particulars may be obtained.

Sheffield.—The Corporation have received the sanction of the Local Government Board to a loan of £68,000 for the extension of Lodge Moor Hospital.

Sible Hedingham.—Mr. N. J. Avey is architect for the proposed new smallpox hospital to be built by the Halstead Rural District Council, North Essex.

Smethwick.—The Joint Hospital Committee have reported to the Town Council that they have approved plans for the extension of the hospital at an estimated cost of £5,783.

Stockton.—The Stockton and Thornaby Hospital Committee are considering additions to the hospital at an estimated cost of £500.

